# Emotional Dysregulation and Time Structure Mediate the Link between Perceived Stress and Insomnia among Unemployed Young People in China: A Cross-Sectional Study

**DOI:** 10.3390/ijerph191911883

**Published:** 2022-09-20

**Authors:** Zhiya Hua, Dandan Ma, Xiaoling Xia

**Affiliations:** 1 School of Government, Shanghai University of Political Science and Law, Shanghai 201701, China; 2 School of Sociology and Political Science, Shanghai University, Shanghai 200444, China

**Keywords:** youth unemployment, unemployed young people, perceived stress, insomnia, emotional dysregulation, time structure, time management, China

## Abstract

In recent years, the problem of youth unemployment in China has become a great concern. Unemployed young people often find themselves under tremendous stress and vulnerable to sleep problems. The link between perceived stress and insomnia has been widely documented, but the potential mechanisms underlying this relationship have not been thoroughly revealed. The objective of this study was to examine the underlying mechanisms linking perceived stress to insomnia through testing the mediating effects of emotional dysregulation and time structure. Through a multiple-stage convenience sampling, 511 unemployed young people (38.6% women; mean age = 21.51; SD = 2.22) were measured using the 10-item Perceived Stress Scale (PSS-10), Insomnia Severity Index (ISI), 16-item Difficulties in Emotion Regulation Scale (DERS-16), and Time Structure Questionnaire (TSQ). Based on the statistical description of the sample, chi-squared tests, bivariate correlation analyses, and mediation analyses were performed. The study indicated that 53.0% (95% CI: 48.7–57.4%) of the participants reported probable insomnia, and both insomnia and perceived stress demonstrated strong associations with emotional dysregulation and time structure, which served as partial mediators between perceived stress and insomnia symptoms according to mediation analyses. Path analysis further revealed that, after controlling for age and gender, emotional dysregulation and time structure accounted for 31.8 and 17.6% of the effect of perceived stress on insomnia, respectively. This study provides empirical support for the association among perceived stress, emotional dysregulation, time structure, and insomnia symptoms. To improve the sleep quality and general wellbeing of unemployed young people, emotional dysregulation and time structure must be taken into consideration.

## 1. Introduction

China’s youth unemployment rate has remained low for many years, but this situation has changed because of the combined effect of slow economic growth and the COVID-19 pandemic. In 2019, China’s youth unemployment rate (16–24 age group) was 12.5% [1]. However, this figure rose to 14.2% in 2020 and climbed to 14.3% in 2021, nearly three times that of the 25–59 age group (4.5%) [1]. In July 2022, it shot up to 19.9%, the highest level on record [2]. A striking feature of China’s youth unemployment is that more and more people with higher education are unable to find work. According to the Ministry of Education of China, more than 20% of college graduates cannot smoothly find work after graduation [3]. Given that China is producing nearly 10 million fresh graduates a year [4], almost two million are expected to join the ranks of the unemployed.

Being unemployed is a stressful life event [5], but those in their late teens and early twenties usually experience more stress, in part because they are making the transition to adulthood [6]. In most societies, finding that first job and gaining economic independence is a sign of a successful, smooth transition [7]. Hence, young people who cannot find work feel added stress and frustration, as well as the stigma of failing to make this transition. Even though they are gradually reaching their physical and psychological maturity, they still lack adequate psychological strategies and social skills to deal with challenges and difficulties [8], and this may enhance their perceptions of stress.

Perceived stress, which is understood to be a person’s subjective appraisal of pressures and stressful situations [9], is an important predictor of health. Although a moderate amount of stress may lead to positive adjustment and good outcomes [10], abundant evidence in the literature points out that excessive stress, whether acute or chronic, causes a variety of health problems [11]. In the same vein, the strong correlation between perceived stress and insomnia has been well documented [12,13,14,15,16]. For example, through an investigation of female college students in the U.S., Lee et al. revealed that high levels of perceived stress were significantly related to sleep difficulties, shortened sleep time, and an elevated level of fatigue [17]. Similarly, the association between stress following unemployment and insomnia symptoms is frequently reported [18,19,20]. For instance, an analysis of data from a five-wave repeated cross-sectional survey in Finland indicated that unemployed respondents reported higher levels of insomnia and stress over time than the employed did [21].

As one of the most common sleep disorders, insomnia refers to difficulty in falling asleep or maintaining sleep, which leads to worse sleep quality, impaired daytime functioning, and personal distress [22]. Although competing pathomechanism models exist, the consensus is that the onset of insomnia has some relationship to hyperarousal [23,24], which can be caused by various variables including physical, psychological, environmental, behavioral, disease, and drug factors [25]. Insomnia is a serious danger to people’s health and general wellbeing. For instance, it is associated with daytime tiredness, lower school achievement, poor work performance, and increases in traffic and workplace accidents [15,26,27]. Moreover, insomnia increases the risk of negative health outcomes in non-communicable diseases [28,29], metabolic diseases [30], and mental health [31,32]. Insomnia is also an important public health problem in China, and the prevalence of insomnia among Chinese adults reached 38.2% in 2021 [33]. Although some studies have explored insomnia and its contributing factors in the general Chinese public, e.g., [34,35], insomnia among unemployed Chinese youth has not received due attention. To prevent the onset of insomnia-related sleep problems among unemployed young people, the processes and mechanisms linking perceived stress to insomnia need further understanding, but these have not been fully revealed.

Many factors contribute to the onset of insomnia, and emotional dysregulation has recently attracted increased interest. According to the cognitive-emotional model of insomnia, emotional dysregulation results in heightened levels of emotional arousal, which in turn affects the balance between arousing and sleep-inducing brain activity and causes sleep disorders [36,37]. Emotional dysregulation is usually defined as difficulties and deficits in the self-regulation of emotions [38]. According to Gratz and Roemer [39], good and adaptive emotional regulation needs four kinds of abilities: recognition and understanding of one’s own emotions, accepting negative moods, effectively managing impulses, and persisting goal-oriented behaviors when experiencing negative emotions, and using situationally dependent emotional regulation strategies. The lack of, or deficiency in, these abilities indicates emotional dysregulation. In recent years, a large number of clinical and subclinical experiences demonstrate that emotional dysregulation and insomnia symptoms are significantly positively correlated [36,40,41]. For example, a study of 1065 college students in China found that those who adopted dysfunctional emotional regulatory strategies such as rumination reported more insomnia symptoms [42], whereas Vandekerckhove and Wang reported that adaptive emotional regulation contributes to good sleep [43]. Moreover, perceived stress may impair emotional regulation abilities and lead to the development of emotional dysregulation. Studies have found that excessive stress may disturb the functioning of the parasympathetic nervous system and impair the performance of inhibitory control, which usually leads to failures in emotional regulation [44]. In addition, emotional dysregulation has been found to be a mediator between perceived stress and health problems such as substance abuse and other additive disorders [45,46,47]. For example, an investigation of 126 people living with HIV/AIDS in Texas revealed that it significantly mediated the association between stress and hazardous drinking [48]. Based on these correlations, emotional dysregulation could mediate the effect of perceived stress on insomnia. However, the extent of this mediation, if any, has not been examined.

Another potential mediator is time structure. Originally proposed by British sociologist Mari Jahoda as a key construct in her latent-deprivation model [49], it has become widely used in the field of unemployment research. After being polished by several researchers, time structure is now understood to mean “the degree to which individuals perceive their use of time as structured and purposeful” [5,50]. According to Feather and Bond, people with higher degrees of time structure use time in a more structured, purposeful manner to ensure goals are met, while those who have lower levels of time structure experience difficulties organizing their lives and setting a routine to achieve goals [51]. Time structure is sometimes viewed as a near synonym for time management [52,53], but there is a slight but crucial difference: time management refers to the ability to order events, organize activities, and allocate time over the course of a day [54]; time structure emphasizes not only the structured and purposeful use of time but also a person’s perceptions of, and feelings about, their use of time [55]. Hence, time structure is a better indicator of the subjective appraisal of time usage. As illustrated by Kelly’s study of a group of undergraduate students [56], time structure, as a kind of mental fact, exerts a more direct effect than time management on psychological activity, behavior, and health status. In fact, a large number of empirical studies, both cross-sectional and longitudinal research, have found that a lower level of time structure is a significant predictor of psychological health problems such as apathy, anxiety, neuroticism, and depression [51,56,57,58]. Other studies have suggested that a higher level of time structure is related to better physical and mental health [59,60], and in recent years, some studies have found that time structure is negatively associated with insomnia symptoms [55,61]. For example, an investigation of 111 participants conducted by Chang and Nguyen reported that the loss of time structure significantly predicted a decline in sleep quality [55]. Conversely, an experimental study of 114 women in China with perimenopausal syndrome revealed that the structured and purposeful use of time improves sleep quality and decreases insomnia symptoms [62]. Other studies reported that perceived stress and time structure were negatively correlated [5,63,64]. For example, a longitudinal study in Kansas of a group of employed, unemployed, and re-employed adults revealed that unemployment stress led to difficulty organizing time effectively and a deterioration in time structure [5]. In addition, research has found that time structure is a significant mediator between some behavioral variables and health [55,63,65]. For example, a cross-sectional investigation conducted by Goodman et al. revealed that time structure mediated the relationship between recreational activities and depressive symptoms among a group of unemployed adults [65]. Hence, based on the aforementioned correlations, we suspect that time structure mediates the effect of perceived stress on insomnia symptoms. However, this mediating model has not been empirically tested.

Summing up, the review of the available literature indicates that perceived stress and insomnia symptoms are significantly correlated, but the underlying mechanisms have not been fully revealed. Meanwhile, the sleep problems of the unemployed young people in China have not received due attention. To fill the gaps in the literature, this study aimed to examine whether emotional dysregulation and time structure mediate the relationship between perceived stress and insomnia symptoms. Specifically, through a cross-sectional investigation of China’s unemployed youth, we tested the following four hypotheses: (1) Perceived stress and insomnia symptoms correlate positively; (2) emotional dysregulation is positively related to perceived stress and insomnia; (3) time structure correlates negatively with perceived stress and insomnia symptoms; and (4) emotional dysregulation and time structure mediate the relationship between perceived stress and insomnia symptoms.

## 2. Materials and Methods

### 2.1. Designs, Sampling Procedures, and Participants

The current study was part of the research on the Unemployed Youth in Contemporary China project. The corresponding author’s institution examined and approved the research protocol. To meet the study objectives, a cross-sectional quantitative research design was adopted. All the data were collected between the winter of 2020 and the spring of 2021 in Shanghai. In stage one, 120 neighborhoods were selected through a stratified convenience sampling. In stage two, our research team received assistance from local NGOs and neighborhood committees to screen the potential participants according to the following inclusion criteria: (1) aged 16–24; (2) not a full-time student nor employed for at least four weeks; and (3) actively looking for work within the past month. In the end, 742 young people met the criteria. Next, our trained research assistants invited them to participate in the research, of whom 578 accepted. In stage three, the research assistants visited the participants at an appointed time and place. Before the formal investigation began, the purpose of the research, the researchers’ obligations to protect the participants’ privacy, and the participants’ rights were gone over in detail. The research assistants especially emphasized that if potential participants chose not to participate, their rights and lives would not be affected. Eventually, 511 unemployed young people volunteered and signed the informed consent forms. They then completed a battery of questionnaires regarding demographic data, unemployment-related information, and psychometric scales.

### 2.2. Variables and Measures

#### 2.2.1. Demographic Information

Several questions regarding the participants’ gender, age, marital status, and unemployment experience were asked to collect information on basic demographic characteristics and unemployment of the participants.

#### 2.2.2. Perceived Stress

We adopted the 10-item Perceived Stress Scale (PSS-10), created by Cohen and Williamson [9], to evaluate the participants’ perceived stress levels. The PSS-10 comprised 10 questions concerning individuals’ feelings and thoughts about their life situations, especially how stressful events affect their lives. The questions were designed on a five-point Likert scale where answers ranged from 0 (“never”) to 4 (“almost always”). The scores of the four negative items (4, 5, 7, and 8) were reversed, and then the scores for all 10 items were added to obtain a total score. A higher total score suggested greater perceived stress. The PSS-10 has been widely used and has indicated good psychometric properties [66,67]. The Chinese version of the PSS-10 has also been validated among Chinese populations [68,69]. In the current study, the Cronbach’s alpha of the PSS-10 was 0.894, suggesting a high internal consistency.

#### 2.2.3. Insomnia

We used the Insomnia Severity Index (ISI), created by Morin [70], to evaluate the participants’ insomnia symptoms. The ISI contained seven questions designed to assess the severity of different facets of insomnia: difficulty initiating or maintaining sleep, trouble waking up early, satisfaction with current sleep pattern, daytime functional impairment, noticeability of impairment caused by sleep problems, and the degree of concern about insomnia. Responses to each item were given on a five-point Likert scale from 0 (“none”) to 4 (“very severe”). The total score ranged from 0 to 28, with higher scores suggesting more severe insomnia symptoms. Studies across the samples showed that the ISI was a brief and efficient measurement tool for evaluating insomnia severity both in clinical diagnosis and sleep disturbance research [71,72]. The ISI has been used among Chinese populations and has indicated good internal consistency and concurrent validity [73,74]. According to the index’s creators, insomnia was considered when the total ISI score was higher than eight. Meanwhile, scores of 8–14, 15–21, and 22–28 were interpreted as subthreshold, moderate, and severe insomnia, respectively [70]. In the present study, insomnia was seen as a categorial variable (with and without) when examining its prevalence, but it was seen as a continuous variable when conducting the correlation and mediation analyses. The ISI indicated high internal consistency in the present study (Cronbach’s alpha = 0.879).

#### 2.2.4. Emotional Dysregulation

Emotional dysregulation of participants was evaluated using the 16 items of the Difficulties in Emotion Regulation Scale (DERS-16) developed by Bjureberg et al. [75]. The DERS-16, adapted from the DERS [39], has shown good psychometric properties [76,77]. The DERS-16 contained 16 self-reported items measuring the deficits in emotional regulation concerning the clarity of emotions, acceptance of negative emotions, access to effective emotion regulatory methods, control of impulsive behavior, and persistence of goal-directed behavior. Responses to each item were designed on a five-point Likert scale from 1 (“almost never”) to 5 (“almost always”). The total score was obtained by summing the scores of 16 items, which ranged from 16 to 80. The higher the total score, the higher the degree of emotional dysregulation. The DERS-16 has been validated among Chinese populations [78], and its internal consistency was satisfactory in the present study (Cronbach’s alpha = 0.837).

#### 2.2.5. Time Structure

We used the Time Structure Questionnaire (TSQ), designed by Bond and Feather [50], to evaluate the time structures of participants. The TSQ comprised of 26 self-reported questions that assessed the extent to which people perceive their time usage as planned, structured, and purposeful according to criteria such as structured routine, activity organization, and sense of purpose (e.g., “Do you have a daily routine which you follow?”; “Do you plan your activities from day to day?”; and “Looking at a typical day in your life, do you think that most things you do have some purpose?”). Responses to each item were given on a seven-point Likert rating scale from 1 (“always”) to 7 (“never”), with higher scores demonstrating higher levels of time structure. The total score, which ranged from 26 to 182, was used in the current study. The TSQ has shown high reliability and validity across various samples, including both unemployed and employed groups [65,79]. A forward and backward translation was conducted for the TSQ. One member of the research team translated the TSQ from English to Chinese, and another member was involved in the translation from Chinese back to English. Discrepancies between the original and translated versions were resolved by consensus. The analysis of consistency indicated that the Chinese version of the TSQ in the current study demonstrated good internal reliability (Cronbach’s alpha = 0.907).

### 2.3. Statistical Strategies

The statistical software used in the analysis was SPSS 26.0 for Windows (SPSS Inc., IBM, Chicago, IL, USA). First, we used descriptive statistics to summarize information on demographic characteristics, perceived stress, emotional dysregulation, time structure, and insomnia symptoms. Second, chi-squared tests were conducted to examine whether a difference in insomnia prevalence existed between groups having different sociodemographic traits. Third, bivariate Pearson correlation analyses were performed to explore the relationships between the main variables. Lastly, mediation analyses were conducted to examine the mediation effects of emotional dysregulation and time structure between perceived stress and insomnia symptoms. Mediation analysis focuses on whether the effects of a proposed independent variable on a dependent variable operate through a proposed mediator. Through examining and testing the underlying operation paths, mediation analysis can reveal the potential mechanisms linking independent and dependent variables. Mediation analysis can be realized by various statistical techniques. In this study, we adopted the bootstrapping method proposed by Preacher and Hayes [80,81] and used PROCESS macro (version 3.5) for SPSS [75] to implement the mediation analyses. According to Baron and Kenny [82], the key to testing mediation effects is to observe whether the regression coefficients (path coefficients) are statistically significant or not. If total, indirect, and direct effects are significant, and the absolute value of direct effect is less than the absolute value of total effect, the proposed mediator partially mediates the relationship between the independent variable and the dependent variable. If total and indirect effects are significant but direct effects are not significant the proposed mediator would be considered a complete mediator. Based on Baron and Kenny’s method, the bootstrapping method can directly compute confidence intervals through a random resampling [80]. Compared to traditional mediation analysis approaches such as causal step regression and structural equation modeling, the bootstrapping test through PROCESS macro not only examines multiple mediators simultaneously but also directly compares the indirect effects of various paths [83,84]. Because our proposed model had two mediators, according to Hayes’ recommendation [83], we used model four in PROCESS macro and adopted 5000 bootstrapped resamples to estimate the mediating effect of emotional dysregulation and time structure on the relationship between perceived stress and insomnia symptoms. The effect would be deemed significant at *p* < 0.05 if the bootstrapped 95% bias-corrected confidence interval did not pass by zero.

## 3. Results

### 3.1. Descriptive Statistics

There were 314 (61.4%) young men and 197 (38.6%) young women in the sample. About three quarters (75.3%) were Shanghai natives. The mean age of the sample was 21.51 (±2.22) years. One hundred and eight people (n = 108), 21.1% of whom belonged to the 16–19 age group, and 78.9% (n = 403) belonged to the 20–24 age group. Most of the participants (87.3%) were not married, and nearly 40% held at least a bachelor’s degree. With regard to the duration of unemployment, 92% of them had been unemployed for more than 3 months. The majority of the participants (72.4%) did not officially register for unemployment.

### 3.2. Prevalence of Insomnia

Among the sampled young unemployed, the prevalence of probable insomnia was 53.0% (271/511) (95% CI: 48.7–57.4%). More precisely, the young unemployed with subthreshold, moderate, or severe insomnia comprised 45.6% (n = 233), 7.2% (n = 37), and 0.2% (n = 1) of the sample, respectively. The results of chi-squared tests (Table 1) suggested that the prevalence of probable insomnia varied according to gender, age group, and duration of unemployment. Specifically, the prevalence of insomnia among unemployed young women was significantly higher than that of young men. Additionally, insomnia prevalence in the 20–24 age group and those who had been unemployed for less than 3 months was higher than that for the 16–19 age group and those who had been unemployed for more than 3 months, respectively. Otherwise, no statistically significant difference was found in insomnia prevalence among the groups.

### 3.3. Correlation Analyses

The main characteristics of the variables of interest are reported in Table 2. The results of the correlation analyses indicated that insomnia and perceived stress correlated positively (r = 0.428, *p* < 0.01). Moreover, a higher level of perceived stress correlated significantly with a higher level of emotional dysregulation (r = 0.406) and a lower level of time structure (r = –0.377). In addition, insomnia was positively associated with emotional dysregulation (r = 0.462), while it correlated negatively with time structure (r = –0.359).

### 3.4. Mediation Analyses

**Figure 1:**
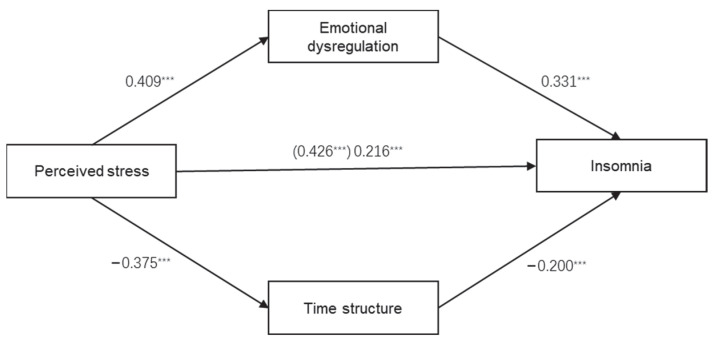
The mediation model for perceived stress, emotional dysregulation, time structure, and insomnia (N = 511, *** *p* < 0.001).

On the basis of the correlation analyses, we applied a multiple mediating model and adopted the bootstrapping technique to estimate the indirect effects of perceived stress on insomnia through emotional dysregulation and time structure. To control for the effect of demographic variables, we treated age and gender as covariates. The result demonstrated that the path coefficient (i.e., standard regression coefficient) from perceived stress to insomnia was 0.426 (*p* < 0.001), suggesting there was a significant total effect of perceived stress on insomnia. Second, the regression analysis results showed that perceived stress had a significant effect on emotional dysregulation (β = 0.409, *p* < 0.001) and time structure (β = –0.375, *p* < 0.001). Furthermore, we discovered significant effects in the pathways from emotional dysregulation and time structure to insomnia (β = 0.331 and –0.200, respectively). Finally, after emotional dysregulation and time structure as proposed mediators were added to the hypothesized model, the direct effect of perceived stress on insomnia was still significant, but its value decreased from 0.426 to 0.216 (see Figure 1). These all indicated that, after controlling for age and gender, emotional dysregulation and time structure partially mediated the link between perceived stress and insomnia.

Moreover, using PROCESS directly calculated the mediating effects of emotional dysregulation and time structure and tested their significance. Because the hypothesized model in the present study has two mediators, we adopted model four in the PROCESS macro. Similarly, age and gender were taken into the model as covariates. Table 3 reports the results of the bootstrapping examination. Because the confidence intervals of all the indirect, direct, and total effects did not contain zero, the mediating effects of emotional dysregulation and time structure between perceived stress and insomnia symptoms were significant.

Furthermore, the results of the indirect, direct, and total effects presented in Table 3 demonstrated that the effect of perceived stress on insomnia decreased from 0.2403 (total effect) to 0.1219 (direct effect) when the two mediating variables (emotional dysregulation and time structure) were taken into the mediation model. Hence, emotional dysregulation and time structure together accounted for 49.3% (0.1184/0.2403) of the total effect. In comparison to time structure (17.6%, 0.0422/0.2403), emotional dysregulation (31.8%, 0.0763/0.2403) contributed 13.1% more to the total effect.

## 4. Discussion

The current study examined the association between perceived stress and insomnia symptoms among China’s unemployed youth through a cross-sectional investigation and tested the possible mediating roles of emotional dysregulation and time structure between perceived stress and insomnia. We found that the prevalence of insomnia in the sample was 53.0%, which was significantly higher than that for young adults (23.2%) [85] and the general public (30.9%) [34] in China. Furthermore, our investigation demonstrated that unemployed young women were more likely to report insomnia symptoms than young men were, which confirmed the previous findings about gender differences in insomnia [86,87,88]. For example, according to Ohayon’s review, women were about 1.4 times more likely than men to report insomnia symptoms [86]. Some researchers argue that this gender difference can be explained by the physical discrepancy between women and men [86]. Given that men and women may also differ in emotional regulation [89] and time usage [90,91], it is necessary to further examine the relationship between insomnia and gender differences in emotion regulation and time usage. It is also noteworthy that unemployed people in the 20–24 age group were more likely to have insomnia symptoms than those aged 16–19. Furthermore, newly unemployed youth (≤3 months) were more likely than long-term unemployed (>3 months) to suffer from insomnia. These findings suggest that China needs to provide its unemployed young people, especially young women, newly unemployed, and those between 20 and 24, with social support and intervention services.

Moreover, the analyses demonstrated that significant correlations existed among perceived stress, emotional dysregulation, time structure, and insomnia symptoms in the young unemployed. First, perceived stress and insomnia correlated significantly. People with higher perceptions of stress were more likely to suffer from insomnia, confirming the stress-induced pathology of insomnia [12]. According to this model, stress may give rise to sleep reactivity and lead to insomnia [92]. Second, more perception of stress was significantly associated with increased emotional dysregulation and a weaker time structure. Hence, the present study provided new evidence for the association between perceived stress and maladaptive behaviors and coping strategies. Third, insomnia symptoms were positively associated with emotional dysregulation but negatively linked to time structure, implying that emotional dysregulation was the vulnerable factor and time structure was the mitigating factor of insomnia-related problems. Consistent with our hypotheses, the aforementioned findings suggest that emotional dysregulation may worsen while time structure may mitigate the effects of stress.

Additionally, as the results of the mediation analyses indicated, emotional dysregulation was a significant mediator between perceived stress and insomnia symptoms, which suggests that those young unemployed who perceive more stress are more likely to experience emotional dysregulation, which in turn contributes to the onset or maintenance of insomnia. This finding confirms previous reports [41,93]. For instance, an investigation conducted by Hoag in a sample of 392 young adult women in Texas found that those who had more negative affect experienced greater difficulty in emotion regulation and poorer sleep quality [93]. Similarly, a cross-sectional investigation of 776 Italian adults conducted by Musetti et al. revealed that emotional dysregulation acted as a mediator between online pornography use and insomnia [94]. Moreover, studies discovered that emotional dysregulation mediated the effect of stress on other psychological disorders such as depression and anxiety [95]. Our study of China’s unemployed youth provided new empirical support for the mediating effect of emotional dysregulation between perceived stress and insomnia, which could perhaps be explained by the hyperarousal theory of insomnia [96]. According to this theory, emotional dysregulation caused by maladaptive emotional reactivity gives rise to persistent autonomic hyperarousal. This breaks the balance between arousing and sleep-inducing brain activity and, in turn, results in sleep problems [36,96]. For example, by investigating the differences between those who suffer from insomnia and those who do not, Palagini et al. found that emotional dysregulation increases the level of the pre-sleep cognitive arousal that leads to insomnia [97].

Furthermore, as hypothesized, the mediation analysis demonstrated that time structure also significantly mediates the link between perceived stress and insomnia. It suggests that unemployed young people with higher levels of perceived stress have difficulty using their time in a structured, purposeful manner, thereby enhancing their risk of insomnia, which accords with previously reported mediating models of time structure [55,57,65]. For instance, Selenko et al. examined the mediation effects of the latent benefits of employment through an empirical study of 1014 participants. They found that time structure partially mediated the effect of employment status on psychological health [57]. Similarly, an investigation of 111 Australian adults––college students, employed workers, and homemakers––reported that time structure partially mediated the association between time management behavior and job satisfaction [55]. One possible explanation of the mediating effect of time structure between perceived stress and insomnia is that it helps maintain a healthy lifestyle and increases good sleep hygiene, which decreases the risk of insomnia [98,99].

To our knowledge, this is the first empirical research on insomnia and its contributing factors among unemployed youth in China. Through a cross-sectional survey, it examined the mediating effects of emotional dysregulation and time structure on the relationship between perceived stress and insomnia. The investigation and the related data reported in this study introduced new evidence to support the conceptual work linking perceived stress, emotional dysregulation, time structure, and insomnia to unemployment. Meanwhile, the findings of this study helped to further understanding of the underlying processes and mechanisms that link perceived stress with insomnia. Moreover, this study tested the mediating effect of time structure between perceived stress and insomnia, which first extended the insight of the latent-deprivation model of unemployment [49] to insomnia-related sleep problems. Additionally, the findings of the present study suggest that cognitive-emotional and behavioral responses to stress have a direct impact on insomnia-related problems, thereby extending the understanding of stress-induced psychopathology [100].

In addition to its theoretical implications, this study has practical value because it revealed the high prevalence of insomnia among China’s unemployed youth. Meanwhile, researchers have recently come to realize that insomnia is not only a symptom of mental health issues but also a catalyst that induces or promotes mental disorders [40]. It is hence of great significance to explore effective approaches to decrease the risk of insomnia. This study determined ways to help unemployed youth reduce stress and lower their risk of insomnia. First, the government can establish special unemployment benefits for unemployed youth; communities can build a more supportive and friendly environment; and families and friends can provide more assistance and care. Second, because emotional dysregulation may be elevated while time structure may reduce the risk of insomnia, they may be seen as targets for clinical intervention. According to the current study, effectively regulating emotions and utilizing time in a structured and purposeful way are helpful to adaptively deal with stressful situations and guarantee sleep quality and quantity during unemployment. Unemployed young people, however, usually lack strategies, methods, or skills to cope with adversity or to manage their emotions and time [101]. Therefore, supportive services should be provided to help them master emotional regulatory strategies and time management skills.

This study, however, had a number of limitations that should be addressed. First, the cross-sectional research design determined that no conclusions about cause-effect between variables could be drawn. Further experimental or longitudinal research is needed to be able to make causal statements. Second, all the subjects were unemployed youth, which may have caused a selection effect. In the field of unemployment research, the selection effect is rooted in the reciprocal relationship between unemployment and impaired health [102]. Hence, it is difficult to determine whether unemployment leads to insomnia or whether insomnia leads to unemployment. To avoid selection effects, future research needs to either investigate employed individuals and make comparisons with the unemployed or adopt a longitudinal research design to investigate people who went from employment to unemployment and those who experienced the reverse. Third, the present study is based on a survey obtained by convenience sampling. Hence, the representativeness of the sample is insufficient, so future studies could use better sampling designs and investigate more segments of unemployed youth. Fourth, the sample consisted of more men (61.4%), which may affect the research results. Future research needs to recruit more women to ensure gender balance. Fifth, insomnia in this study was measured exclusively by a self-reporting questionnaire, and the study failed to distinguish between transient sleep disturbance and chronic insomnia, as is occasionally highlighted by some researchers [103]. Both of these may have led to overestimating the prevalence of insomnia. In future research, cross-disciplinary cooperation should be considered to measure insomnia. Meanwhile, it would be interesting to make a comparison between the objective quality of sleep and subjective complaints among people with insomnia. Sixth, this study did not consider the impact of depression. In fact, depression was not only a significant predictor of sleep problems [104], but also an important factor that can influence people’s emotional regulation and time usage [62,105]. Therefore, future research needs to take depression into account and examine the complicated interactions between depression, insomnia, emotion regulation, and time structure. Finally, because this research was a cross-sectional study based on a small sample obtained by convenience sampling, the generalizability of its findings is limited. Meanwhile, emotional dysregulation and time structure only exerted a partial mediation effect on the relationship between perceived stress and insomnia, which implies that there may be other mediators. Hence, further investigation is needed to better understand the underlying mechanisms linking perceived stress to insomnia.

## 5. Conclusions

This study explored the prevalence of insomnia in unemployed young women and men in China and found that insomnia-related sleep problems in China’s unemployed youth are a concern. Meanwhile, this study provided an in-depth examination of the operational mechanisms linking perceived stress to insomnia symptoms by revealing the mediating effects of emotional dysregulation and time structure. Additionally, path analysis demonstrated that emotional dysregulation as a mediator contributes more to the indirect effect than time structure does. These findings not only extend the academic understanding of stress-related health problems such as insomnia but also provide a basis for preventing and intervening in insomnia-related sleep problems.

## Figures and Tables

**Table 1:** Demographic information of participants and the results of chi-squared tests.

Demographic Characteristic	%	N = 511	Insomnia (ISI ≥ 8)		*p* (Chi-Squared Test)
			Yes (n = 271)	Prevalence (row %)	
Gender					
Men	61.4	314	143	45.5	<0.001
Women	38.6	197	128	65.0	
Age group (years old)					
16–19	21.1	108	48	44.4	0.044
20–24	78.9	403	223	55.3	
Place of household registration					
Shanghai	75.3	385	203	52.7	0.809
Non-Shanghai	24.7	126	68	54.0	
Educational level					
Primary school and below	1.2	6	3	50.0	0.152
Middle school	20.9	107	48	44.9	
High school (including secondary vocational school)	40.1	205	106	51.7	
University	36.2	185	108	58.4	
Graduate school	1.6	8	6	75.0	
Marital Status					
Unmarried	87.3	446	231	51.8	0.251
Married	12.5	64	39	60.9	
Divorced or others	0.2	1	1	100.0	
Unemployment registration					
Registered	27.6	141	78	55.3	0.523
Not registered	72.4	370	193	52.2	
Duration of unemployment					
1 month<~≤3 months	8.0	41	28	68.3	0.041
					
					
					
>3 months	92.0	470	243	51.7	

Note: The level of significance of chi-squared test was set at *p* < 0.05.

**Table 2:** Main characteristics of variables of interest and correlation coefficients between main variables.

Main Variables	Range	Min	Max	Mean	SD	1	2	3	4
1. PS	0–40	5	38	23.10	7.51	-			
2. ED	16–80	26	74	50.85	7.99	0.406	-		
3. TS	26–182	36	122	81.44	17.20	–0.377	–0.226	-	
4. Insomnia	0–28	0	22	7.91	4.23	0.428	0.462	–0.359	-

Note: PS = perceived stress, ED = emotional dysregulation, TS = time structure, Min = minimum values, Max = maximum values, SD = standard deviations. All the correlations are significant at the level of 0.01.

**Table 3:** Indirect, direct, total effects, and 95% confidence intervals for the mediation model.

No.	Pathways	Effect Value	95% CI	
			Lower	Upper
1	PS—ED—Insomnia	0.0763	0.0539	0.1012
2	PS—TS—Insomnia	0.0422	0.0258	0.0607
3	PS—Insomnia (Direct effect)	0.1219	0.0749	0.1688
4	Total effect	0.2403	0.1959	0.2848

Note: CI = confidence intervals, PS = perceived stress, ED = emotional dysregulation, TS = time structure.

## Data Availability

The data presented in this study are available on request from the corresponding author. The data are not publicly available to preserve the participants’ privacy.
